# FUS BBBO with a Portable FUS System and real time mapping

**DOI:** 10.1002/alz70856_103290

**Published:** 2025-12-26

**Authors:** Elisa Konofagou

**Affiliations:** ^1^ Columbia University, New York, NY, USA

## Abstract

**Background:**

Treatment of neurological diseases has been at the forefront of medical research for more than a century with limited success due partly to the blood‐brain barrier (BBB) that impedes both delivery and biodistribution of the drug delivered. Focused ulltrasound (FUS) methodologies in conjunction with systemically administered microbubbles have been shown capable of transcranially and transiently opening the BBB over the past two decades. More recently, those efforts have resulted into clinical translation in a variety of brain diseases such as brain tumors and neurodegenerative diseases such as Alzheimer's (AD) disease. Most FUS systems that have been reported for AD treatment are either confined in the MRI or use invasively implanted transducers. Our group has pioneered a portable system for opening the BBB at the patient's bedside safely and efficiently, i.e., within 20‐30 min with real‐time cavitation mapping.

**Method:**

Five AD patients underwent FUS with microbubbles for transcranial BBB opening in their prefrontal cortex. Real‐time cavitation mapping system was also implemented to transcranially map the cavitation occurrence and dose in real time throughout the BBB opening procedure in Alzheimer's disease (AD) adult and diffuse intrinsic pontine glioma (DIPG) pediatric patients. Passive acoustic mapping (PAM) with coherence factor (CF) correction is used to passively map the microbubble activity within the brain. Multi‐element CF‐PAM allows us to determine the exact location of the BBB opening. The system was first optimized in targeting in non‐human primates followed by feasibility in the prefrontal cortex in AD patients, achieving feedback rates of 2 Hz during the clinical procedures.

**Result:**

It was found that 60% of the patients exhibited beta amyloid reduction as evidenced by the SUVr reduction on PET imaging. We also found that cavitation dose can inform the beta amyloid reduction with 80% correlation between the cavitation dose and SUVRr reduction observed.

**Conclusion:**

A portable FUS system was found to be safe and efficacious in reduction of beta amyloid in patients with mild to moderate AD. Real‐time information on cavitation can provide feedback on both the safety and efficacy of treatment. Future studies will investigated effects on cognition and tau reduction.

